# Molecular Dynamics Simulation for the Effect of Fluorinated Graphene Oxide Layer Spacing on the Thermal and Mechanical Properties of Fluorinated Epoxy Resin

**DOI:** 10.3390/nano11051344

**Published:** 2021-05-20

**Authors:** Qijun Duan, Jun Xie, Guowei Xia, Chaoxuan Xiao, Xinyu Yang, Qing Xie, Zhengyong Huang

**Affiliations:** 1 Hebei Provincial Key Laboratory of Power Transmission Equipment Security Defense, North China Electric Power University, Baoding 071003, China; duan_ncepu@163.com (Q.D.); xgw_97@163.com (G.X.); XCX@ncepu.edu.cn (C.X.); yang_HD@126.com (X.Y.); xq_ncepu@126.com (Q.X.); 2 State Key Laboratory of Alternate Electrical Power System with Renewable Energy Sources, North China Electric Power University, Beijing 102206, China; 3 State Key Laboratory of Power Transmission Equipment & System Security and New Technology, Chongqing University, Chongqing 400044, China; huangzhengyong@cqu.edu.cn

**Keywords:** fluorinated epoxy resin, fluorinated graphene oxide, ordered filling, molecular dynamics, elastic modulus, glass transition temperature, microscopic parameters

## Abstract

Traditional epoxy resin (EP) materials have difficulty to meet the performance requirements in the increasingly complex operating environment of the electrical and electronic industry. Therefore, it is necessary to study the design and development of new epoxy composites. At present, fluorinated epoxy resin (F-EP) is widely used, but its thermal and mechanical properties cannot meet the demand. In this paper, fluorinated epoxy resin was modified by ordered filling of fluorinated graphene oxide (FGO). The effect of FGO interlayer spacing on the thermal and mechanical properties of the composite was studied by molecular dynamics (MD) simulation. It is found that FGO with ordered filling can significantly improve the thermal and mechanical properties of F-EP, and the modification effect is better than that of FGO with disordered filling. When the interlayer spacing of FGO is about 9 Å, the elastic modulus, glass transition temperature, thermal expansion coefficient, and thermal conductivity of FGO are improved with best effect. Furthermore, we calculated the micro parameters of different systems, and analyzed the influencing mechanism of ordered filling and FGO layer spacing on the properties of F-EP. It is considered that FGO can bind the F-EP molecules on both sides of the nanosheets, reducing the movement ability of the molecular segments of the materials, so as to achieve the enhancement effect. The results can provide new ideas for the development of high-performance epoxy nanocomposites.

## 1. Introduction

Epoxy resin reacts with a curing agent to form a polymer with a three-dimensional network structure. The cured product has excellent electrical insulation, mechanical properties, and chemical corrosion resistance. Therefore, EP materials are widely used in electrical insulation, electronics, aerospace, machinery, and construction [1,2,3,4]. As the operating conditions of epoxy resin materials become increasingly complex, higher requirements have been put forward for their thermal, mechanical, and insulation properties [5,6]. In recent years, fluorinated epoxy resin (F-EP) has gradually attracted researchers’ attention due to excellent insulation and dielectric properties coming from the extremely strong electronegativity of fluorine and the high bond energy of C-F bond [7,8,9]. However, the mechanical strength, heat resistance, and thermal conductivity of F-EP materials cannot meet the requirements of high voltage insulation materials, which has become a key factor restricting its further application. Therefore, it is necessary to improve its thermal and mechanical properties.

Nano modification is one of the significant means to improve the properties of epoxy composites. With the continuous advancement of the research and application of carbon nanomaterials, modified graphene materials doped with epoxy resin have become a hot spot in current research [10,11,12,13]. Graphene oxide is a graphene-based material containing a large number of oxidizing functional groups obtained after graphite is oxidized. It retains most of the excellent properties of graphene and has high surface activity. However, due to the strong van der Waals force [14] on the surface of graphene-based materials, graphene oxide flakes in the composites are easy to form serious aggregates. Relevant experimental studies also confirmed that the distribution of graphene directly affects the macro properties of the composites [15,16,17]. It is found that the dispersion of nano fillers in polymer matrix can be improved by fluorination modification, and the fluorine-containing groups will form a shielding layer on the surface of nano materials, so as to inhibit the agglomeration of fillers [18,19,20]. This provides a method guidance for the construction of a modified graphene packing network with good dispersion.

One dimensional or two-dimensional nano fillers are often affected by their own spatial structure in many aspects. However, the agglomeration and disordered distribution of nano fillers will seriously affect the modification effect [21,22,23]. The researchers found that reasonable assembly of fillers in the polymer matrix has better modification effect on the properties of composites [24,25,26]. In addition, the physical and chemical properties of nano fillers have an important influence on the cross-linking structure and crystallization behavior of polymers, but the mechanism has not been revealed, and the research on this problem is rare. Sanat K. Kumar et al. reviewed the outstanding theoretical research progress of polymer-nanoparticle hybrids, focusing on the functionalization of nanomaterials and self-assembly methods of fillers, and pointed out that the design of the nanofiller network is a key point of future research [27]. Professor Ahmad Jabbarzadeh has conducted in-depth and systematic research on the crystallization behavior of nanocomposite polymers. The effects of the shape, size, and volume fraction of fillers on the crystallization behavior of the polymer were analyzed, and the crystallization mechanism of the nanocomposite polymer was revealed [28,29]. These studies provide strong support for the development of high-performance functional nanocomposites. The two-dimensional properties of modified graphene make it easy to construct the filler network with layered structure [30,31]. However, there is still a gap in the research on the ordered filling of functional graphene nanosheets in epoxy resin materials. Moreover, it is difficult to fix the space position of modified graphene by experimental method, so molecular simulation technology can be used to realize the pre-study of this method.

At present, molecular dynamics (MD) simulation technology has been widely used in the design, development, and performance analysis of materials [32,33,34,35]. The MD technology can be used to simulate the structure and properties of high molecular polymers from the molecular scale, and analyze the correlation between the microstructure and macroscopic properties of the polymer. MD technology has been widely used by researchers in the development and research of epoxy composite materials, which not only saves research time and economic costs, but also deepens the understanding of the modification mechanism of epoxy composite materials [36,37,38]. Shenogina [39] built a diglycidyl ethers bisphenol A (DGEBA)/diethyltoluenediamine (DETDA) cross-linking network system through MD simulation technology, and studied the influence of the number of molecules, molecular chain length, and cross-linking degree on the thermal performance of the cross-linking network. Wang [40] et al. used MD simulation to modify graphene with functional groups, and explored the effect of graphene modification methods on the Young’s modulus and thermal conductivity of composites. The results showed that the carboxyl and amine functionalized graphene nanocomposites have optimal performance, and the numerical results are in good agreement with the experimental data.

In this study, we established a model of fluorinated graphene oxide (FGO)/F-EP composites with layer spacing of 3 Å, 6 Å, 9 Å, 12 Å, and disorderly filling. Based on molecular dynamics simulation technology, the effects of different distribution characteristics of FGO nanosheets on the thermal and mechanical properties of the composites were studied. This study provides a new theoretical understanding for epoxy resin reinforced by modified graphene, which can guide the design and development of high-performance epoxy nanocomposites.

## 2. Model Construction and Simulation Calculation

**Figure 1:**
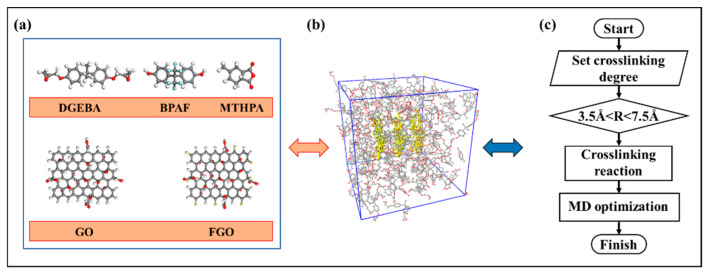
Establishment of molecular models. (**a**) Monomer molecular models of DGEBA, BPAF, MTHPA, GO, and FGO. (**b**) Model of F-EP filled with FGO. (**c**) Crosslinking procedure.

In this study, we used Material Studio 7.0 to complete the modeling and calculation. Firstly, the cross-linking network model of fluorine-containing epoxy resin was constructed, and the DGEBA was fluorinated with hexafluorophobia bisphenol A (BPAF) as a fluorinating agent [8,41]. The monomer molecular models of DGEBA, methyltetrahydrophthalic anhydride (MTHPA), and BPAF were constructed. Each monomer molecule was labeled with reactive atoms and optimized in MD geometry, as shown in Figure 1a. The optimized monomer molecules were put into an amorphous periodic box according to the ratio in Table 1. The box density was set at 0.6 g/cm^3^, the model temperature was 600 K, and the model was dynamically optimized. Furthermore, graphene unit cells were introduced, and graphene supercells were constructed according to the periodic box size. The molecular model of graphene oxide (GO) monomer was constructed based on Lerf–Klinowski method, and the simplest molecular formula was C_10_O_1_(OH)_1_(COOH)_0.5_. In the model, epoxy groups and hydroxyl groups are randomly attached to the surface, while carboxyl groups are distributed at the edges. In this paper, a GO sheet containing 82 carbon atoms and 22 oxygen atoms is constructed to represent graphene oxide, and hydrogen atoms are added to prevent unsaturated edges. The GO model is shown in Figure 1a. Then, the graphene oxide is fluorinated, and fluorine atoms are manually added at its edges to construct a FGO model.

**Figure 2:**
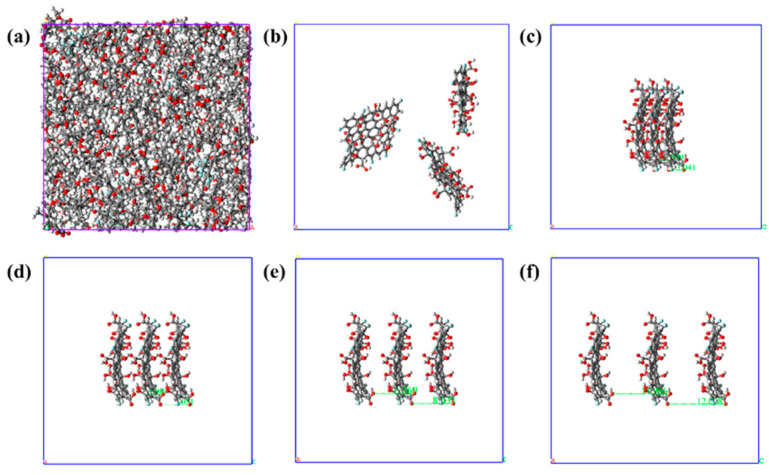
Molecular models. (**a**) Unfilled F-EP. (**b**) F-EP with disorderly filled FGO nanosheets. Figure (**c**) to (**e**) are the F-EP composite models with ordered filling of FGO (epoxy resin molecules are hidden). The layer spacing of each model is as follows: (**c**) 3 Å, (**d**) 6 Å, (**e**) 9 Å, (**f**) 12 Å.

The FGO nanosheets were filled into the F-EP box to construct the composite material model, as shown in Figure 1b. The molecular dynamics calculation results of epoxy resin are greatly affected by the number of model molecules, so we control the same number of molecules in each model, and only change the interlayer spacing of modified graphene nanosheets. We filled three FGO Nanosheets in the cell and controlled the FGO Nanosheets array to be located in the center of the cell. The distance between FGO layers was set as 3 Å, 6 Å, 9 Å, and 12 Å, respectively. The position coordinates of FGO molecules in the model were fixed, and a disordered filling model was constructed as the control group. The unfilled F-EP model and the FGO filled composites models with hidden epoxy resin are shown in Figure 2.

The geometric optimization of the composite model with different FGO layer spacing was carried out. The box with the lowest energy was selected to calculate the binding energy parameters of GO, FGO, and F-EP. After the nano-filler is doped into the epoxy matrix, an inorganic−organic interface layer will be formed between filler and matrix. Generally, the stronger the interfacial force between the nano-filler and the matrix, the better the performance of the corresponding composite. Binding energy is an important parameter to characterize the interfacial bonding force between modified filler and epoxy system. The larger its absolute value, the stronger the interaction force between matrix and filler in composite material [42], and the calculation formula is as follows:

(1) $$E_{\mathrm{interface}}=E_{\mathrm{total}}-E_{\mathrm{fiber}}-E_{\mathrm{resin}}$$

where E_interface_ is the interfacial bonding energy between nanofiller and matrix, E_total_ is the total energy of composite material, E_resin_ is the energy of epoxy substrate, and E_fiber_ is the energy of nano-filler. The calculation results of bonding energy are shown in Table 2.

As illustrated in Table 2, the interfacial bonding energy between FGO and matrix after grafting fluorine element is obviously improved compared with GO. The analysis shows that fluorine has strong electronegativity, and bonding with carbon will make the common electrons of fluorocarbon atoms tend to fluorine atoms, forming a negative charge shielding layer, which inhibits the agglomeration effect of modified graphene materials and provides more surface area for the interaction between filler and substrate. In addition, due to the strong polarity of C-F bond, it is easy to react with groups in epoxy resin, and the interfacial bonding strength between filler and epoxy matrix can be enhanced at low filling mass fraction. Therefore, the fluorinated grafting modification of graphene oxide nanosheets is beneficial to improve its dispersibility and compatibility in epoxy resin matrix.

The model of composites with cross-linking degree of 90% was obtained by further running the cross-linking program [4], the calculation program is shown in Figure 1c. The cross-linking temperature was set at 600 K, and the truncation radius of reaction atom bonding was set at 3.5 Å−7.5 Å. After cross-linking, the model was optimized geometrically and MD treated to eliminate the internal stress generated during cross-linking. In MD process, NVT of 100 ps and NPT relaxation of 200 ps were carried out at 600 K, the Andersen and Berendsen were chosen to control temperature and pressure, respectively. The pressure of molecular dynamics process is 10^5^ Pa and the time step is 1 fs. The size of the cross-linking network model of the optimized epoxy resin composite is 43 Å*43 Å*43 Å. The optimized composite models were annealed, with the annealing span controlled at 600–280 K and the annealing rate selected as 20 K/100 ps. After each round of annealing, NPT optimization treatment of 200 ps was performed to eliminate the internal stress caused by temperature change. Finally, the epoxy composite models at different temperatures were output for the subsequent calculation of system performance and structural parameters.

## 3. Results and Discussion

### 3.1. Static Mechanical Performance Calculation

In this paper, the static constant strain method was used to analyze the elastic mechanical properties of the system [43]. After MD optimization process, the system had reached the mechanical equilibrium. Then, a small strain was applied to it and the energy optimization was carried out again. In the process of molecular simulation, the strain is applied to different directions and repeated many times. The stiffness matrix can be calculated as the second derivative of the deformation energy (U) per unit volume (V) with respect to strain (ε).

(2) $$C_{\mathrm{ij}}=\frac{1}{V}*\frac{\partial^{2}U}{\partial \varepsilon_{i}\partial \varepsilon_{j}}$$

In this process, three groups of uniaxial tension, three groups of uniaxial compression, and six groups of shear deformation were applied to the balanced epoxy resin system in x, y, and z directions respectively. The stiffness constant matrix can be obtained by extracting the stress of the optimized deformation system. We found that the elastic mechanical properties of the matrix did not show obvious anisotropy, which is related to the small size and mass fraction of FGO. Therefore, we calculated elastic mechanical parameters according to the isotropic stiffness matrix of conventional epoxy composites.

(3) $$C_{\mathrm{ij}}=\left[\begin{array}{cccccc} \lambda +2\mu & \lambda & \lambda & 0 & 0 & 0\\ \lambda & \lambda +2\mu & \lambda & 0 & 0 & 0\\ \lambda & \lambda & \lambda +2\mu & 0 & 0 & 0\\ 0 & 0 & 0 & \mu & 0 & 0\\ 0 & 0 & 0 & 0 & \mu & 0\\ 0 & 0 & 0 & 0 & 0 & \mu \end{array}\right]$$

where λ and μ are elastic constants, and the corresponding constants can be obtained by further solving the matrix.

(4) $$\left\{ \begin{array}{l} \lambda =\frac{1}{6}\left(C_{12}+C_{13}+C_{21}+C_{23}+C_{31}+C_{32}\right)\\ \mu =\frac{1}{3}\left(C_{44}+C_{55}+C_{66}\right) \end{array}\right.$$

According to λ and μ, the static elastic modulus of the system such as Young’s modulus E, shear modulus G, and bulk modulus K can be obtained.

(5) $$\left\{ \begin{array}{l} E=\mu \frac{3\lambda +2\mu }{\lambda +\mu }\\ G=\mu \\ K=\lambda +\frac{2}{3}\mu \end{array}\right.$$

**Figure 3:**
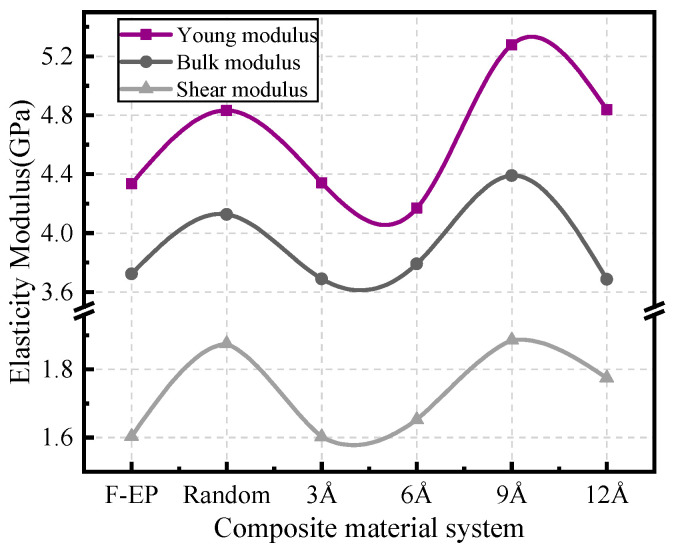
Static elastic modulus of FGO/F-EP composites with different distribution characteristics.

In this study, the static elastic modulus of each model at 300 K is calculated, and the results are shown in Figure 3. It can be found that when the interlayer spacing is changed from 3 Å to 12 Å, the mechanical properties of the material show a sinusoidal-like behavior of first decreasing and then increasing. When the spacing between FGO layers is 9 Å, the static elastic modulus of the composite reaches the highest, and it can be considered that the comprehensive mechanical properties of the composite are better at this time. It is considered that when the interlayer spacing is small, the structure distribution of the composite material is uneven, which cannot utilize the characteristics of high elastic modulus of modified graphene. However, when the interlayer spacing is too large, the van der Waals interaction force between FGO and epoxy matrix is weak, which leads to the weak deformation resistance of the system. After adjusting the distance between the layers, the modified graphene can fully contact with the matrix material and form a well dispersed adsorption layer. The external stress on the substrate can be effectively buffered in the adsorption layer and transferred to the modified graphene sheet with excellent mechanical properties. At the same time, the force of FGO on the matrix also limits the movement of a polymer molecular chain, which makes the epoxy resin form a relatively close cross-linking network. This can also better bear the stress of the material. Therefore, reasonable control of FGO filler network structure can improve the mechanical properties of the composites.

### 3.2. Thermal Performance Analysis

#### 3.2.1. Glass Transition Temperature

**Figure 4:**
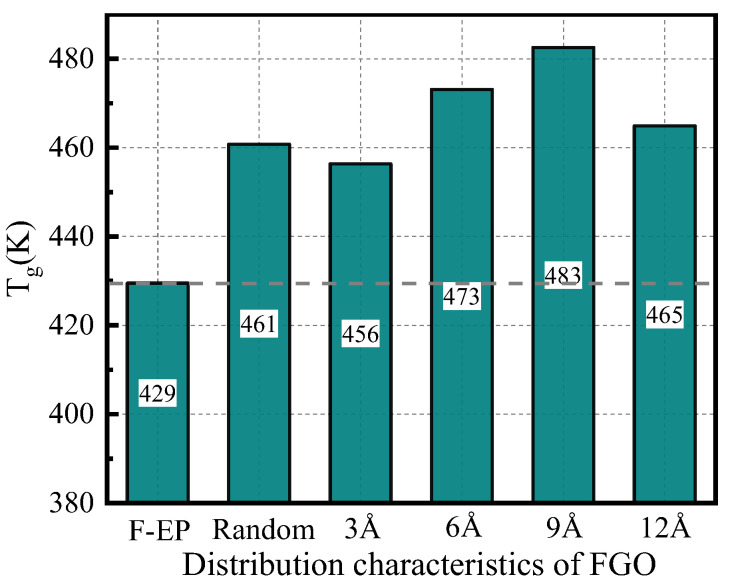
Glass transition temperature of FGO/F-EP composites with different distribution characteristics.

Glass transition temperature (T_g_) can be calculated by extracting the density and temperature parameters of epoxy composites during annealing. During the transition from glassy state to the rubbery state of polymer, the density and volume of materials will change with the increase of temperature. There are obvious differences between the change rates of density and volume of materials with temperatures before and after the glass phase transition [44]. According to this rule, we can get the T_g_ of epoxy composites by fitting the temperature-density curve, and the change of T_g_ of FGO/F-EP with different interlayer spacing, as shown in the Figure 4. It can be found that, similar to the change of static elastic modulus, T_g_ also shows a sinusoidal change with the increase of the interlayer distance. The T_g_ value reaches the highest when the interlayer distance is 9 Å, which is 483 K. We believe that when the interlayer spacing is small, FGO exists in epoxy substrate in a form similar to agglomerated nano-filler, which has an adverse effect on the improvement of T_g_ of epoxy composites. With the increase of spacing between FGO layers, FGO nanosheets with fixed spatial positions form a localized filler network with pivotal effect, and F-EP is attracted and fixed near the filler to a certain extent, forming a relatively stable cross-linked network structure. Meanwhile, fluorine atoms existing in both filler and matrix provide a more stable acting force for this connection form, which delays the glass phase transition process of the composites and improves T_g_. Similarly, when the interlayer spacing is too large, the pivotal effect of the filler is weakened, and the advantages of the filler itself and the network structure regulation cannot be fully exploited, which makes the T_g_ a downward trend.

#### 3.2.2. Coefficient of Thermal Expansion

**Figure 5:**
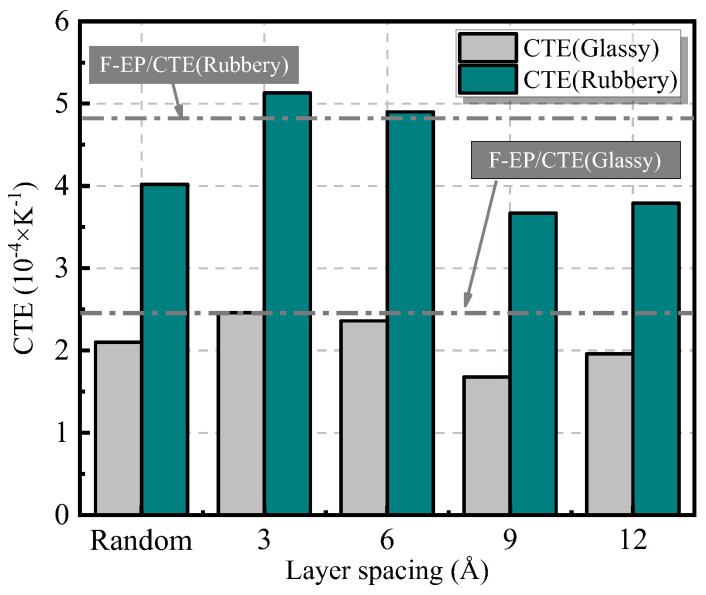
Coefficient of thermal expansion of FGO/F-EP composites with different distribution characteristics.

Coefficient of thermal expansion (CTE) is an important parameter to characterize the stability of polymer materials at high temperature, which can be calculated by calculating the internal stress generated after the internal deformation of the system [45]. The calculation formula is:

(6) $$\mathrm{CTE}=\frac{1}{V_{0}}{\left(\frac{\partial V}{\partial T}\right)}_{P}$$

where V_0_ is the volume of the initial model after cross-linking (in this study, the volume parameter of the system at 300 K is used), and P is the standard atmospheric pressure. According to the formula (5), ${\left(\frac{\partial V}{\partial T}\right)}_{P}$ can be obtained by linear fitting the volume and temperature parameters of the model. Furthermore, the CTE of FGO/F-EP epoxy composites with different interlayer spacing can be obtained, and the results are shown in the Figure 5. It can be found that the overall CTE of the composite material system after adding FGO is lower than that of the pure resin system. However, when the distance between the FGO layers is near the range of 3 Å~6 Å, the filler spacing is too small, resulting in agglomeration. At this time, the filler lacks binding effect on the epoxy resin. As the temperature increases, the heated movement of the filler makes agglomerates expanded, which leads to a slight increase in the thermal expansion coefficient of the composite material. When the interlayer spacing of FGO nanosheets is about 9 Å, the two-dimensional nanosheets have a greater binding effect on the polymer segments and are not affected by filler agglomeration. When the composite material is thermally expanded, the segments preferentially fill the free volume space, so the thermal expansion coefficient of the system is small.

#### 3.2.3. Thermal Conductivity

**Figure 6:**
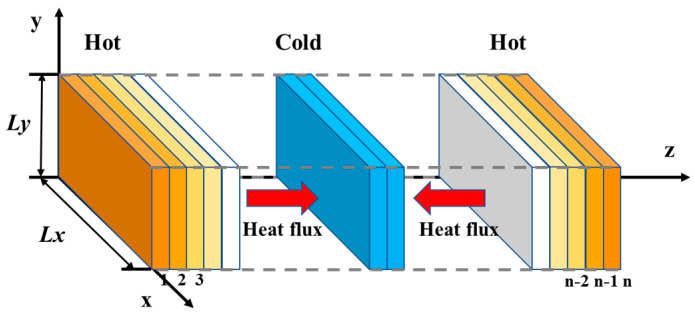
Mechanism model for heat conduction calculation of RNEMD.

In order to study the influence of FGO interlayer distance on the axial thermal conductivity (TC) of materials, the TC of different systems was studied by using TC script. In this script, the TC is calculated according to reverse perturbation nonequilibrium molecular dynamics (RNEMD), and its computer model is shown in Figure 6 [46]. The calculation formula of thermal conductivity is:

(7) $$\kappa =-\frac{\sum_{\mathrm{transfers}}\frac{m}{2}\left(v_{h}^{2}-v_{c}^{2}\right)}{2tL_{x}L_{y}\langle \partial T/\partial z\rangle }$$

where κ is the exchange rate of hot particles, v_c_ is the exchange rate of cold particles, L_x_L_y_ is the area where heat transfer occurs, and $\frac{\partial T}{\partial Z}$ is the temperature gradient in the z direction.

**Figure 7:**
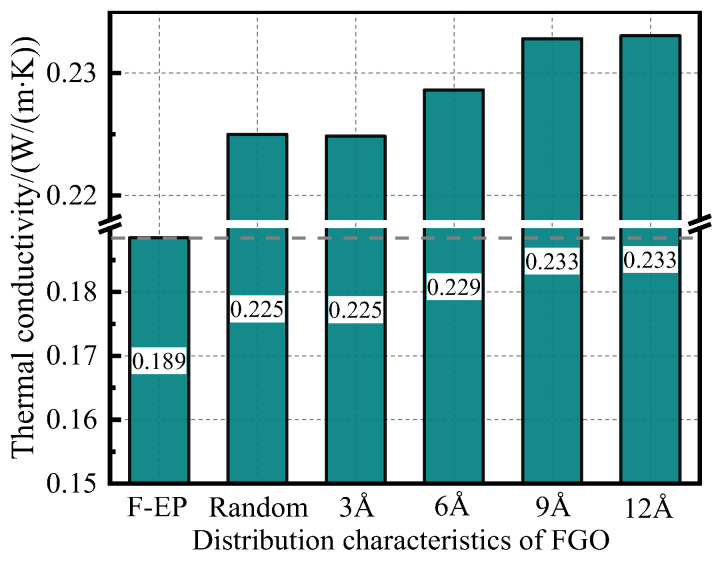
Thermal conductivity of FGO/F-EP composites with different distribution characteristics.

In order to improve the accuracy of the simulation calculation, the composite material model needs to be extended three times in the *Z*-axis direction before calculating the TC to obtain a 1 × 1 × 3 composite unit cell [47]. The TC of each system is calculated 10 times and the average value is taken. The results are shown in the Figure 7 below.

Comparing the TC data of different distribution states of FGO/F-EP system, it can be seen that the orderly-filled FGO can greatly improve the TC of the F-EP system. When the distance between the modified graphene layers is greater than 9 Å, the TC of the system is increased by about 4% relative to the disordered filler distribution system. This shows that graphene itself has good TC, but the commonly used disorderly doping methods often make fillers agglomerate and stack together, which reduces the interaction area between fillers and matrix. At the same time, there is a lack of binding force for the polymer segments outside the filler interface, which makes it difficult to overcome the interfacial thermal resistance between the filler and the matrix in the heat transfer process. As a result, even if the nano filler is doped with high thermal conductivity, the improvement of the thermal conductivity of the composite is very limited.

After FGO is filled in an orderly manner, the uniformly dispersed two-dimensional FGO nanosheets provide a sufficient surface area of the filler, and the presence of fluorine also greatly enhances the bonding between the filler and the matrix. In addition, the polymer segments are bound by spatially fixed fillers, which makes the cross-linking network structure of epoxy resin molecules between nanosheets more compact. These factors play a bridging role for F-EP segments on both sides of the filler, which will greatly reduce the interfacial thermal resistance during heat conduction, thus forming an effective heat conduction network and improving the overall thermal conductivity of the composites. At the same time, the interconnected network structure formed between FGO and matrix relies on strong interaction force, and evenly distributed adsorption layers are produced. When the temperature rises, the heat flow can spread more rapidly along the uniform adsorption network structure, thus significantly improving the TC of epoxy composite materials. Based on this, it can be predicted that the thermal conductivity of composite materials can be significantly improved by designing a reasonable spatial structure of the filler network and increasing filler concentration.

### 3.3. Microscopic Parameter Calculation and Principle Discussion

In order to analyze the influence mechanism of the above performance changes, the free volume, mean square displacement (MSD), and axial density distribution were further calculated.

#### 3.3.1. Free Volume

According to the free volume theory, the total volume (V_t_) of solid or liquid substances can be divided into occupied volume (V_0_) and free volume (V_f_) [48]. The size of the free volume in polymer composites has a significant impact on the thermal and mechanical properties of the material. By analyzing the distribution of free volume inside the material, the mechanism of the change in the macroscopic properties of the material can be explored. In this study, due to the MD treatment during model construction, the volume of different epoxy systems is not exactly the same. Therefore, the V_f_ of each epoxy system cannot be directly compared. Consequently, the percentage is used to calculate the fractional free volume (FFV) to characterize; its expression is [49]:

(8) $$\mathrm{FFV}=\frac{V_{f}}{V_{0}+V_{f}}\times 100\%$$

**Figure 8:**
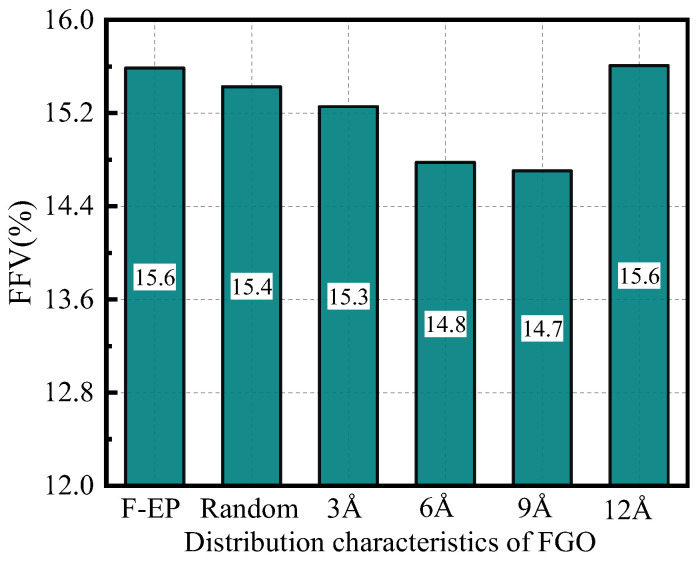
FFV of FGO/F-EP composites with different distribution characteristics.

This paper calculated the FFV of FGO/F-EP system with different interlayer spacing at 300 K, and the results are shown in Figure 8. With the continuous increase of the distance between the FGO filler layers, the FFV shows a trend of first decreasing and then increasing. Compared with the random system, when the interlayer spacing is 3 Å to 9 Å, the FFV of the composite material is relatively reduced, and when the interlayer spacing is further increased, the FFV of the system increases significantly. We think that FGO with random distribution is easy to agglomerate in the system, which makes the free volume of the system relatively large, while the FGO with reasonable interlayer spacing will be evenly filled in the epoxy system, and the binding effect of FGO on both sides of the matrix material will lead to the orderly arrangement of epoxy molecules, which will reduce the FFV of the system. When the layer spacing is further increased, FGO is distributed too loosely in the composite material, and the binding effect on the epoxy resin molecules is relatively weakened. At the same time, more holes are generated in the interface area, which makes the FFV significantly increased.

#### 3.3.2. Mean Square Displacement

**Figure 9:**
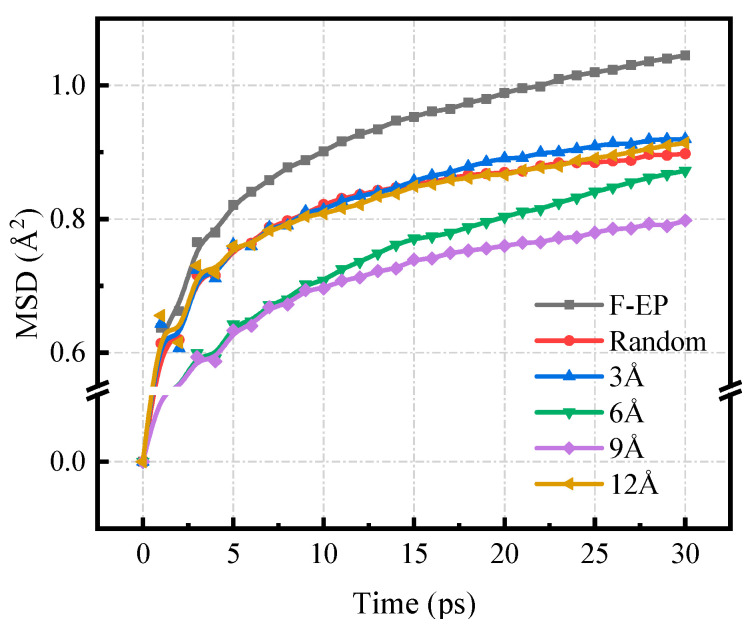
MSD of F-EP composite systems with different FGO layer spacing.

A large number of studies have shown that the movement of molecular segments in polymer composites is one of the important factors affecting material properties. The strength of segment movement is directly related to the degree of looseness of the composite network structure. In many application scenarios, reducing the movement capacity of the chain segment is a key method to ensure that the material has high mechanical properties. The strength of the internal chain segment movement of the material can be characterized by the MSD parameter [50], and the MSD is defined as:

(9) $$\mathrm{MSD}=\frac{1}{3N}\sum_{i=0}^{N-1}\left({\left|R_{i}\left(t\right)-R_{i}\left(0\right)\right|}^{2}\right)$$

where N is the total number of atoms in the system, ***R***_i_(t)and ***R***_i_(0) respectively represent the displacement vector of the atom i in the epoxy system at time t and the initial time. This paper calculated the MSD parameters of the FGO/F-EP system with different interlayer spacing at 300 K, and the results are shown in Figure 9. It can be seen from the figure that the order of MSD of F-EP with different FGO distribution is 9 Å < 12 Å < Random < 6 Å < 3 Å, and this result is generally consistent with the trend of the FFV. The analysis suggests that the filler itself has an inhibitory effect on the movement of the epoxy resin molecular chain, but the excessively agglomerated filler has little effect on the matrix, which causes its effect on the epoxy molecular chain to be limited. After FGO is filled in order, a binding layer can be generated in the system through interaction force. At the same time, the fluorine-containing group can also bond with the epoxy matrix. The strong polar C-F bond also forms a certain barrier effect, and further guides the regular distribution of the resin matrix chain segments, thereby effectively reducing the MSD of the system.

#### 3.3.3. Axial Density Distribution

**Figure 10:**
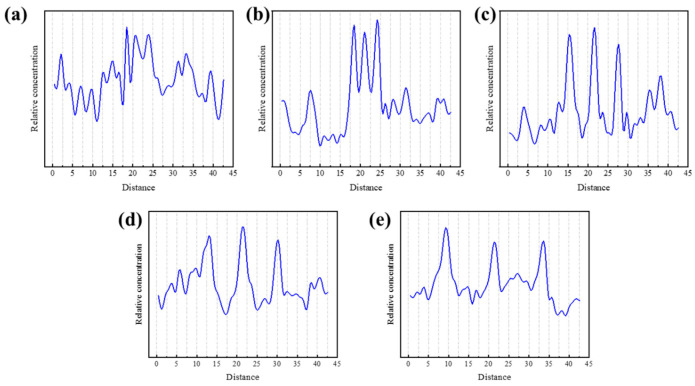
Axial density distribution of FGO/F-EP composites with different distribution characteristics. (**a**) Random; (**b**) 3-FGO/F-EP; (**c**) 6-FGO/F-EP; (**d**) 9-FGO/F-EP; (**e**) 12-FGO/F-EP.

In order to study the effect of graphene addition on the distribution of resin matrix molecules in the composite material, we calculated the relative density distribution curve of the FGO/F-EP composite material model in the axial direction (perpendicular to the FGO surface). The long distance of epoxy resin along the z-axis is divided into several small areas. The results of axial density distribution are shown in Figure 10.

When FGO is disordered, the axial density of the system shows strong fluctuation, and there is no obvious density concentration area. When FGO is filled orderly, the axial density distribution curve of the epoxy composite material model shows three sharp peaks, and the peak position is the z-axis coordinate corresponding to the three intercalated FGO in the system. It can be found that the density of the system near the FGO nanosheets is relatively high, and with the increase of the interlayer spacing, the peak width gradually increases, which indicates that the epoxy resin molecules during the cross-linking reaction will be bound on both sides of the FGO. This is also in line with the previous forecast. The 3 Å model can clearly see the overlap of the peaks, which proves the effect of filler agglomeration. The overlap effect of the 6 Å model is weakened, but the peak width does not change significantly. The peak width of the 9 Å model is significantly increased, and the adsorption effect of the FGO nanosheets on the epoxy molecules can be clearly seen. When the interlayer spacing is further increased, the peak amplitude and peak width in the axial density curve decrease, which is believed to be the reason that the excessive distance between the FGO layers leads to the weakening of the binding effect on the epoxy resin molecules.

Based on the research results, we believe that the pre-fixed two-dimensional filler network can be used to control the cross-linking network of epoxy resin materials. After functionalizing graphene by fluorination, the epoxy resin can be induced to cross-link into bonds. The network structure of epoxy resin can be controlled by the binding effect of the filler network, so that the regular cross-linking region can grow orderly along the spatial structure of filler network. Through this idea, the number of free segments in polymer materials can be effectively reduced, and the movement ability of the segments can be reduced, so as to improve the mechanical strength of the material and increase the glass transition temperature. This will greatly expand the application of polymer composites and provide theoretical guidance for the design of high-performance polymer nanocomposites.

## 4. Conclusions

In this paper, the model of ordered filled FGO nanosheets was constructed, and the effect of FGO nanosheets spacing on the thermal and mechanical properties of fluorinated epoxy resin composites was studied. It shows that the orderly filling of FGO can significantly improve the static elastic modulus, T_g_, CTE, and TC of the composite materials. The interlayer spacing distribution of FGO nanoflakes in the matrix also has an obvious effect on the thermodynamic properties of the composites. The results show that the composite has the best comprehensive properties when the interlayer spacing of FGO is about 9 Å. Among them, Young’s modulus, bulk modulus, and shear modulus are increased by 9.2%, 6.36%, and 0.57%, respectively. The glass transition temperature increases by 21.79 K, the thermal conductivity increases by 3.47%, and the thermal expansion coefficient of glass state decreases by 20%.

Furthermore, the influence mechanism of FGO interlayer distance on the properties of the composites was analyzed by calculating the microscopic parameters of the systems. After ordered filling, the aggregation characteristics of FGO are weakened, and the fillers can be evenly distributed in the composites. There is a strong interaction between the fluorinated FGO and F-EP, which can induce the ordered distribution of epoxy resin molecules on both sides of the two-dimensional filler and cross-linking. This makes the FFV of the materials significantly reduced, and the chains segment motion capacity is also limited by the filler network. When the FGO layer spacing is too large, the binding effect is weakened, which leads to the disorder of the microstructure of the composites. By analyzing the axial density distribution of the composite material, it is found that the fixed-space functionalized filler network structure does have an adsorption effect on epoxy resin molecules, and can bind them on both sides of the two-dimensional filler to guide its orderly cross-linking. We predict that there is a key connection between the crystallization behavior and glass transition behavior of epoxy resin materials. In the future, we can control the cross-link behavior of polymer materials by designing functionalized two-dimensional filler networks and develop high-performance polymers composite materials.

## Figures and Tables

**Table 1:** Composition of FGO modified F-EP Composites.

System	Layer Spacing (Å)	Number of Molecules			
		FGO	DGEBA	MTHPA	BPAF
F-EP	–	0	50	100	25
Random	–	3	50	100	25
3-FGO/F-EP	3	3	50	100	25
6-FGO/F-EP	6	3	50	100	25
9-FGO/F-EP	9	3	50	100	25
12-FGO/F-EP	12	3	50	100	25

**Table 2:** Binding energy of different modified graphene/F-EP systems (kcal/mol).

System	E_resin_	E_fiber_	E_total_	E_interface_
GO/F-EP	−4761	−769	−4157	1373
FGO/F-EP	−4761	−805	−3766	1799

## Data Availability

Data is contained within the article.
